# Enhanced Erosion–Corrosion Resistance of Tungsten by Carburizing Using Spark Plasma Sintering Technique

**DOI:** 10.3390/ma13122719

**Published:** 2020-06-15

**Authors:** Yan Jiang, Junfeng Yang, Zhuoming Xie, Qianfeng Fang

**Affiliations:** 1 International Institute of Vanadium and Titanium, Panzhihua University, Panzhihua 617000, China; jiangyanzky@163.com; 2 Key Laboratory of Materials Physics, Institute of Solid State Physics, Chinese Academy of Sciences, Hefei 230031, China; zmxie@issp.ac.cn

**Keywords:** tungsten, W–Cr–C coating, carburization, intergranular corrosion, pitting corrosion

## Abstract

The biggest obstacle for the application of tungsten as the target materials in the spallation neutron source is its serious corrosion in the coolant of flowing water. For this reason, W–Cr–C clad tungsten was developed by tungsten carburizing in a spark plasma sintering device, with superior corrosion resistance in the static immersion and electrochemical corrosion test. This work focused on its erosion and corrosion performance in a flowing water system, based upon test parameters simulated under the service conditions. W–Cr–C clad tungsten showed superior corrosion resistance to that of bare tungsten due to the corrosion form changing from the intergranular corrosion of bare tungsten to pitting corrosion of W–Cr–C coating. The corrosion rate of tungsten was as high as tenfold that of the coated sample at 20 °C, and at most fourfold at 60 °C after testing for 360 h. Effects of water velocity and temperature on pitting and intergranular corrosion were investigated in detail and their corresponding corrosion mechanisms were analyzed and discussed.

## 1. Introduction

Tungsten is being chosen as the most favorable spallation target material for the spallation neutron source (SNS) due to its obvious advantages including high melting point, high neutron yield, and superior thermal conductivity [1,2]. However, on the issue of compatibility with flowing water coolant, tungsten has long been challenged because of its serious corrosion in water, especially in the case of flowing water where both erosion and corrosion occur simultaneously [3,4,5,6].

To improve the corrosion resistance, tungsten cladding with high temperature and corrosion resistance coatings have been developed as a target material such as HIPed tantalum-clad tungsten [7,8]. However, it suffers from issues of interface defects [7] and the high decay heat of tantalum [8,9]. Alternatively, coatings such as tungsten carbide and chromium carbide have been generally adopted to protect the substrate from corrosion under harsh environments in engineering and industrial fields [10,11,12,13,14,15]. In the conventional preparation of cemented carbides, cobalt as binder phase is commonly introduced to densify the particles, which is known as a poorly resistant element to corrosion [16]. However, the development of binder-free tungsten carbide coatings needed to solve two problems: density and brittleness. To this end, carburizing by spark plasma sintering (SPS) technology was employed to produce tungsten carbide coatings on tungsten [17]. SPS is a high-temperature and fast-process sintering technique that provides a fast heating/cooling rate, short consolidation time, and controllable pressure, having been widely used in densification fabrication of nanomaterials, gradient functional materials, and ceramic materials. By SPS, the carburized layer over 20 μm thick was quickly achieved at 1600 °C, holding for 10 min with a pressure of 45 MPa [17], and a double thick layer was available when 20 min and 30 min were tried. On this basis, W–xCr–C (x = 0.5, 1, 2, 3, and 6 wt.%) composite layers with higher compactness and uniform grain size distribution were fabricated in the same way [18]. Electrochemical corrosion measurements on a WC clad tungsten system and W–Cr–C clad tungsten system indicated that the W–1%Cr–C clad tungsten sample performed well and exhibited the lowest corrosion current density [19].

As a result of these findings, W–1%Cr–C (W–Cr–C for short in the following text) clad tungsten by the SPS method was chosen as the potential material to undergo the following dynamic corrosion test in flowing water; flowing water (pH: 6~8) was adopted as the coolant in service condition to remove the heat in the target chamber. According to the simulation for the 100 kW neutron spallation source, a flow velocity of about 2 ms^−1^ is enough for the heat to be removed from the target, under which the highest target temperature is around 60 °C [2,20]. Therefore, this work investigated the corrosion performance of W–Cr–C clad tungsten at such conditions with the main purpose to explore and understand the behavior and mechanism in the erosion–corrosion process. Bare tungsten was taken into comparison.

## 2. Materials and Methods

**Figure 1:**
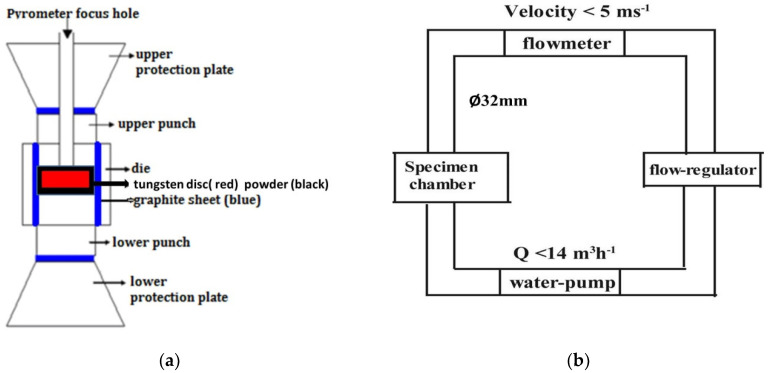
The schematic diagram of the sample placement in the spark plasma sintering setup (**a**) and the self-designed water flowing device (**b**).

A commercial grade tungsten disc (density: 18.48 g/cm^3^, Zhuzhou Cemented Carbide Group Co. Ltd., Zhuzhou, China; the main elements included are listed in Table 1) with cylindrical form (Ø16 mm × 3 mm) was embedded in the mixed powder of graphite (99.95%, 8000 mesh, Aladdin) and 1 wt.% chromium (99.5%, ≥325 mesh, Aladdin, Shanghai, China), and then placed in a graphite mold and sintered in a SPS furnace (HPD 5, FCT Systeme GmbH, Rauenstein, Germany), as shown in Figure 1a. A target temperature of 1600 °C holding for 10 min with a pressure of 45 MPa was exploited on the graphite die (Ø20 mm). The diameter of the die could change from 10 mm to 100 mm according to the sample dimensions. Pressure was used to bring the powder and disc into full contact. The heating and cooling rates of SPS were both 100 °C/min. After sintering, the disc was taken out and cleaned ultrasonically in acetone. A scanning electron microscopy (SEM)(Sirion 200, FEI, Hillsboro, OR, USA) and electron backscatter diffraction (EBSD) detector (Aztec Nordlys Max3, Oxford Instruments, Oxford, UK, incorporated in the SEM device) was applied to characterize the surface morphology, phase composition, and distribution. The samples for the EBSD measurements were mechanically polished with W0.5 diamond paste and then electropolished in 2% NaOH solution. Phase identification was derived from the crystal structure database (HKL, ICSD, and NIST) installed on the EBSD system.

The erosion–corrosion experiments were conducted in a home-made pipe flow circulating system as illustrated in Figure 1b. Diameter of all water passages was 32 mm. The water-pump was placed into an improved thermostatic water-container (0.6 × 0.6 × 1 m) where the water circulated. PH of the water was about 6~7. The oxygen content in the passages can be regarded as a saturation value since the water-container is a semi-open vessel. The tested samples were side by side fixed on the inner upper wall of the specimen chamber, leaving only one exposed surface (Ø16 mm) parallel to the flow direction.

Table 2 lists the test parameters with reference to the simulated service conditions. The flowing velocity and temperature was controlled by the flow regulator and the thermostatic bath, respectively. The error is also marked out at Table 2. The temperature value 20 °C was the entrance temperature of the flowing water. At the relative low temperature of 20 °C, a test at the largest velocity of 2 ms^−1^ was conducted, in light of the small difference in the corrosion behavior between 1 and 2 ms^−1^. After the experiments, the corrosion performance was evaluated by analyzing the corrosion rate and the corroded morphology.

The weight was recorded every 12 h in the erosion–corrosion test, using a balance with an accuracy of 0.01 mg. The average corrosion rate  ν (g/cm^2^·h) was calculated from the following equation:

(1) $$\nu =(m_{0}-m_{t})/s\cdot t$$

where $m_{0}\;$ is the original mass of specimen, ${\;m}_{t}$ the mass of specimen after corrosion for t hours, s (cm^2^) the exposure area before corrosion and t (h) the corrosion time. As the change in s after corrosion is very small, it is considered as a constant. It is worth pointing out in the gravimetric measurements that it was not easy to accurately clean up the corrosion deposits of the corroded specimens without damaging the matrix. Therefore, after the tests, the specimens were directly dried in a vacuum oven at 100 °C for 12 h without any surface treatment, and then their mass $m_{t}$ was measured. If ν > 0, it means a mass loss and removal of the corrosion products from the surface. According to the variation of the ν value, one can judge what happened to each specimen in the corrosion process. The corroded surface morphology was studied by means of SEM observations.

## 3. Results

### 3.1. Morphology and Phases

**Figure 2:**
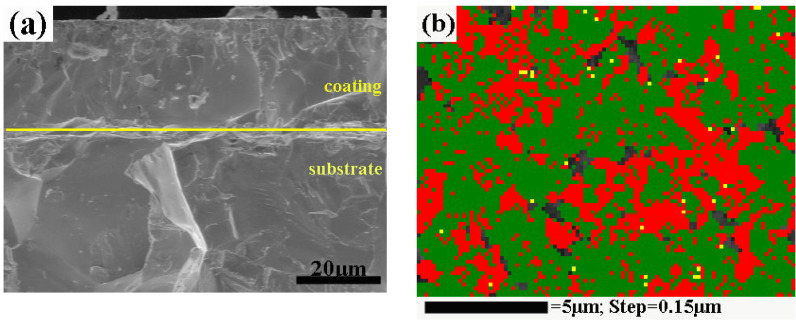

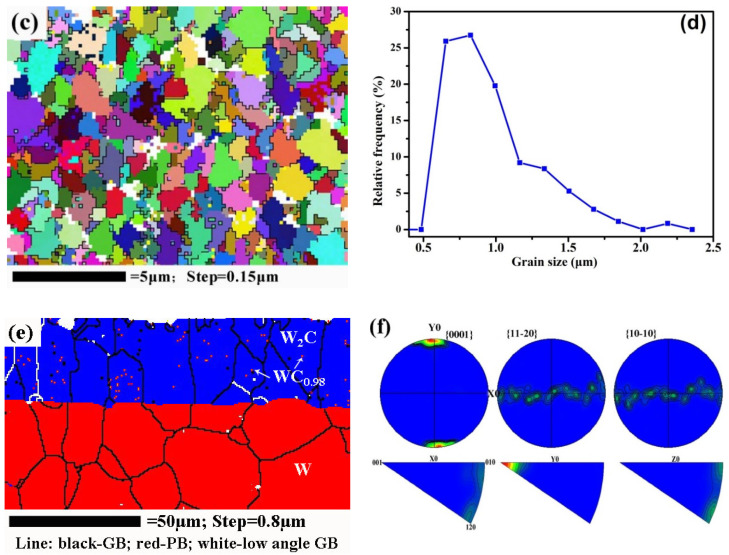
(**a**) Scanning electron microscopy (SEM) image of the cross-section of coating; (**b**) phase distribution of the coating surface (an indexing rate of about 91%, the black refers to the unindexed sites, the red to the WC phase, the green to WC_0.98_, the yellow to Cr_7_C_3_); (**c**) Euler image and (**d**) grain size distribution of the WC and WC_0.98_ phases; (**e**) phase distribution of the inner coating (the blue refers to the W_2_C phase, the red to the W substrate, the pink dots are the WC_0.98_ phases; GB: grain boundary; PB: phase boundary); (**f**) pore figure and inverse pore figure of the inner coating.

Before corrosion measurement, the morphology, compositions, and phase distribution of the coated sample were characterized by the EBSD system. The fractured surface of the coatings showed good bonding with the substrate and high compact structures with a uniform thickness of around 25 μm (Figure 2a). The coating consisted of external W–Cr–C (hexagonal WC (ICSD [15406]) and WC_0.98_ (ICSD [77738]) plus dispersed Cr_7_C_3_ (ICSD [52289]) (Figure 2b,c) and intermediate hexagonal W_2_C (ICSD [77567]) (Figure 2e). Close to the carbon and chromium-rich zone, tungsten carbide reached stoichiometry WC (nonstoichiometric WC_0.98_ is also commonly designated as WC) with an average grain size of 800 nm (Figure 2c,d), and chromium carbide is indexed as Cr_7_C_3_. Along the diffusion path, the composition becomes the carbon-depleted W_2_C and nearly no chromium carbides are detected in the intermediate layer due to the preferential diffusion of carbon. The W_2_C phase accounts for more than 95 vol.% of the coating due to its much lower Gibbs free energy than WC at high temperature [21]. Furthermore, a small fraction of WC_0.98_ precipitates were found in the W_2_C grains (Figure 2e), which were derived from the partial solid-state decomposition of W_2_C below 1250 °C [22]. The vast majority of the W_2_C phase remained stable due to the fast cooling rate (100 °C /min). Figure 2f shows the pole figures and inverse pole figures with respect to the growth direction of the W_2_C layer. Interestingly, the W_2_C layer presented obvious crystallographic preferred orientations: the <001>//Y axis (the sintering pressure direction). Strong fiber texture <001> suggests the formation of a columnar structure of W_2_C coating via spark plasma sintering.

Such a gradient structure from base to bottom by carburizing through SPS technology could release thermal stress and avoid cracks in the coating. Other technology like HVOF (high velocity oxygen fuel) spray can also be applied in creating nano WC-based coatings made of powders [23]. The spray process acts like the high temperature sintering of the raw powders, and micro-cracks are caused by thermal stress in the spray process. More importantly, like other conventional preparations of a WC coating, the bonding phase of cobalt is commonly introduced to densify the WC, which is known as in-resistant element to corrosion. 

### 3.2. Corrosion Rate

**Figure 3:**
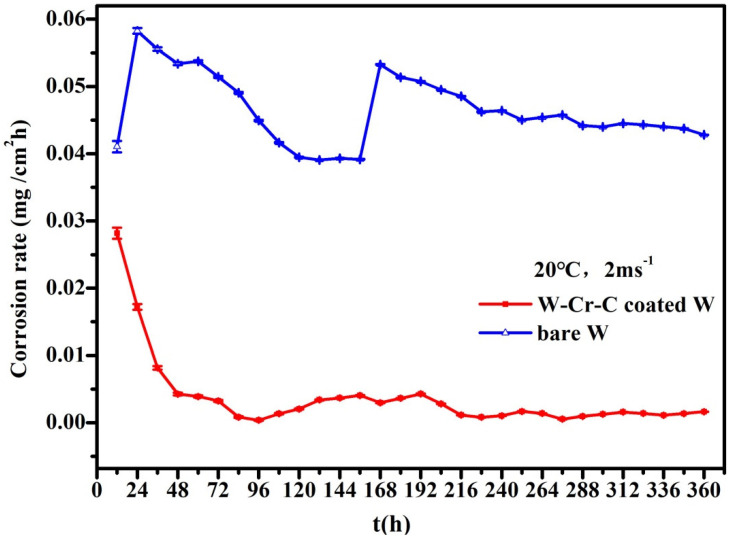
Variation of the corrosion rate with time for samples at the velocity of 2 ms^−1^ under the water temperature of 20 °C.

Under a water temperature of 20 °C, the corrosion rate as a function of time is shown in Figure 3. In general, the corrosion rate for tungsten clad with W–Cr–C decreased with increasing time and gradually went to a steady state after many fluctuations. The fluctuations imply that the accumulation and breakaway of the corrosion products occurred alternatively, namely coexistence of chemical and mechanically driven corrosion. The remarkable maximum corrosion rate belongs to bare tungsten, which was located uppermost over the entire time range and finally reached as high as tenfold that of the coated sample, which was relatively stable around the zero line.

**Figure 4:**
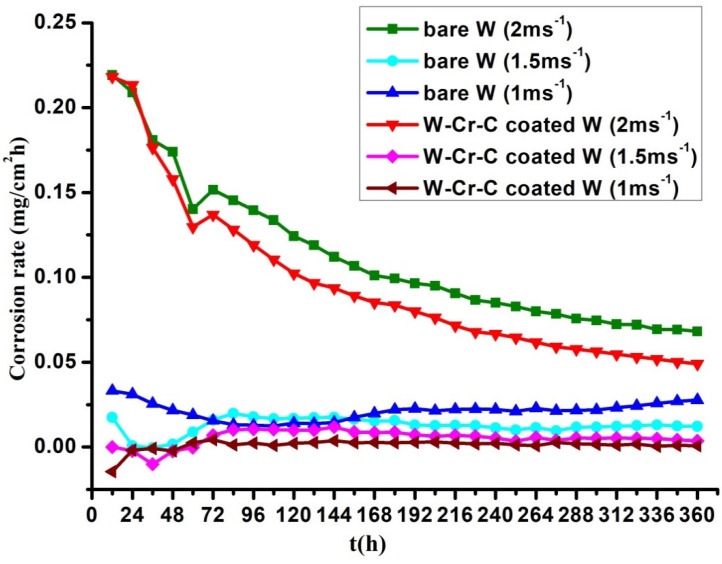
Variation of corrosion rate with time for samples at various flow velocities under the water temperature of 60 °C.

The corrosion rates under the water temperature of 60 °C are shown in Figure 4. The change in corrosion rate was positively related to the flow rate. When the flow velocity was 1 ms^−1^ and 1.5 ms^−1^, the corrosion rate of the W–Cr–C coated W sample was less than 0.01 mg·cm^−2^·h^−1^. The corrosion rate of bare tungsten sample almost doubled and its fluctuation with time was larger. When the flow velocity increased to 2 ms^−1^, the corrosion rates of all samples increased to higher values, varying in the range of 0.25–0.05 mg·cm^−2^·h^−1^ during the experiment time of 360 h.

### 3.3. Corrosion Mechanism

**Figure 5:**
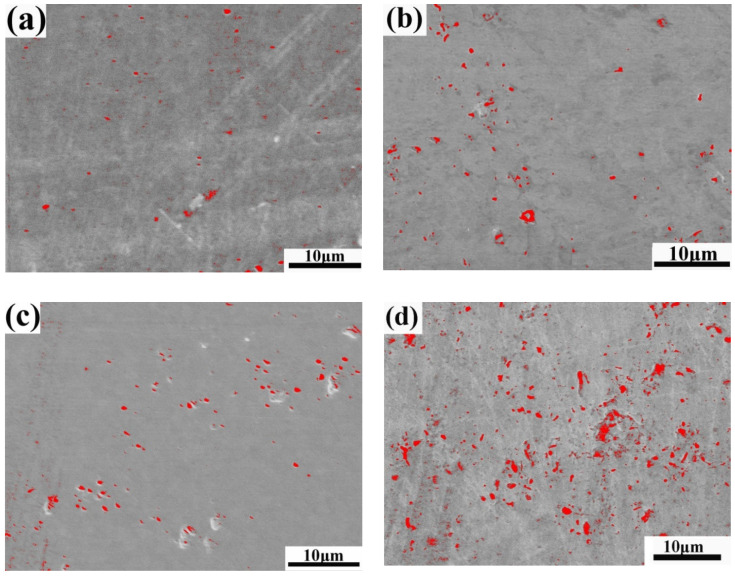

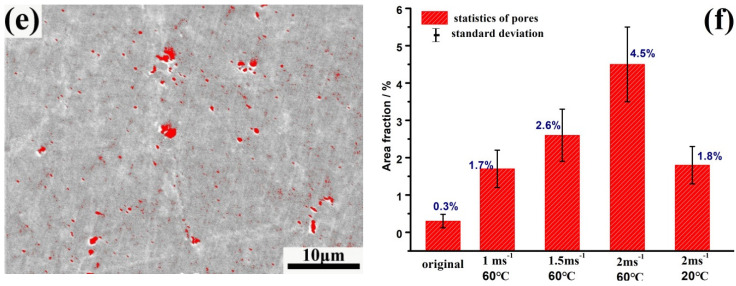
SEM image of the W–Cr–C coated tungsten samples (the red refers to the pores): (**a**) before corrosion; (**b**) corrosion at 1 ms^−1^, 60 °C; (**c**) corrosion at 1.5 ms^−1^, 60 °C; (**d**) corrosion at 2 ms^−1^, 60 °C; (**e**) corrosion at 2 ms^−1^, 20 °C; and (**f**) the bar chart of pitting fraction with velocity and temperature.

The corroded appearances of tungsten clad with W–Cr–C are shown in Figure 5. The initial surface of the W–Cr–C coating (Figure 5a) presented deficiencies of small scattered pores left by SPS fabrication. After the 360 h test, the morphology changed slightly, but increased pores with increasing velocity or temperature, displaying a dominant feature of pitting corrosion (Figure 5b–e). Depth and the number of pores are difficult to quantify, so Figure 5f counts the area fraction of pores using image software (based upon the difference in primary contrast of images) by collecting six SEM graphs with the same magnification to describe the changes of pitting. Under the water temperature of 60 °C, the area fraction of the pores increased from the initial 0.4 ± 0.1% up to 4.5 ± 1% when the flow velocity varied from 0 to 2 ms^−1^. However, there was no single linear or parabolic law between the pore area fraction and flow velocity for a remarkable rise at the flow velocity of 2 ms^−1^. When the water temperature dropped to 20 °C, the reduction in pore area fraction reached 60%, almost close to the rate of temperature decline. Pitting area dependent upon velocity and temperature agreed well with the corrosion rate curves.

**Figure 6:**
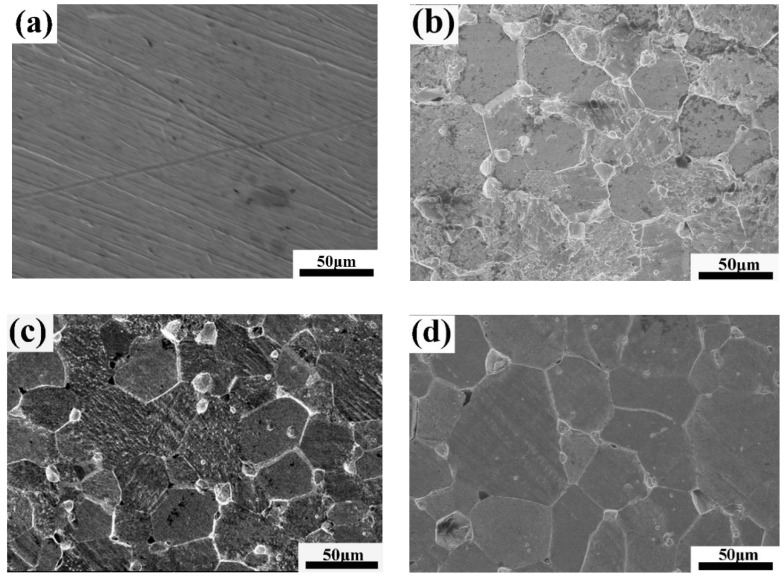

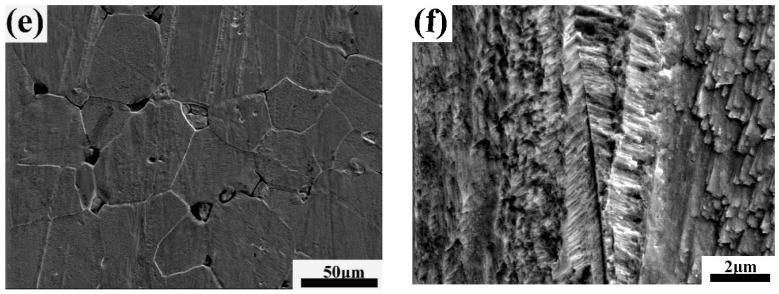
SEM images of bare tungsten: (**a**) before corrosion; (**b**) corrosion at 1 ms^−1^, 60 °C; (**c**) corrosion at 1.5 ms^−1^, 60 °C; (**d**) corrosion at 2 ms^−1^, 60 °C; (**e**) corrosion at 2 ms^−1^, 20 °C; and (**f**) corrosion on grains.

The corrosion behavior of bare tungsten was totally different, as shown in Figure 6. A rough metallographic structure with bimodal grain-size distribution appeared as a consequence of remarkable intergranular corrosion (Figure 6b–e) as well as slight dissolution of the tungsten grains (Figure 6f). The intergranular corrosion at high flow velocity (2 ms^−1^) was serious, accompanied by the formation of deep groove and micro corrosion pits caused by grain exfoliation, indicating that a mechanical effect would promote intergranular corrosion, weakening the GBs cohesion and loosening the GBs, especially at the triple junction.

## 4. Discussion

Based upon the above results, the corrosion rate of W–Cr–C clad tungsten is much lower than that of bare tungsten due to the corrosion form changing from the intergranular corrosion of bare tungsten to the pitting corrosion of W–Cr–C coating. Pitting corrosion is a unique form of anodic reaction in the electrochemical reaction as well as a local corrosion. It causes less weight loss, so the change in corrosion rate with time gradually moves to a relatively steady state, as shown in Figure 3 and Figure 4. The occurrence of pitting corrosion is known to be closely related to the environment and surface morphology and composition of materials. The medium used in this test containing special ions of oxygen and chlorine become the necessary trigger for pitting corrosion. In the service condition, chlorine and oxygen concentrations need to be controlled to reduce the pitting corrosion. Furthermore, reducing the porosity of W–Cr–C coating by fine polishing will help decrease the nucleation sites of pitting.

Occurrence of the intergranular corrosion on tungsten is essentially induced by the impurities, as listed in Table 1. It was already understood that the impurities were prone to segregate at grain boundaries (GBs), which resulted in a large composition difference between the GBs and tungsten grains. The nonmetal impurities such as phosphorus, silicon, oxygen, nitrogen, and their compounds acted as intergranular embrittlement elements and mild acidic electrolyte. The impurities iron, aluminum, and silicon, due to lower potential than tungsten, became the anode, which constituted a corrosion cell with tungsten around and induced pitting corrosion at the GBs. With the exacerbation and development of pitting corrosion, it evolved into macroscopic corrosion along the GBs, namely electrochemically-driven intergranular corrosion. In the regions away from GBs, chemically driven dissolution of tungsten proceeds through the interaction with oxygen and water. Understandably, the dissolution rate at the grain boundary is much higher than that on the tungsten grains. Temperature has an accelerated effect on both types of dissolution.

Intergranular corrosion at the triple junction was much more remarkable. From the perspective of defects, the volume percentage of the GBs and defect density at a triple or multiple-junction are much larger than that at conventional GBs, so corrosion would occur more easily at such regions. From the aspect of interface energy, the corrosion behavior of fine grains at the trigeminal boundaries is analogous to the pinning behavior of the second particles occupying similar sites of matrix. The pinning interaction of the second particles with the matrix grains depends on the contact area of the particles with boundaries. The smaller the second particle, the less the boundary area is occupied. For particles with a smaller size, there is a stronger unpinning tendency of the boundaries because of the lower decreased interface energy [24]. Once the pits at the triple junction of GBs are produced, it leads to a noticeable change in weight. This accounts for the large fluctuations of the corrosion rate for tungsten.

Regardless of whether it is intergranular corrosion or pitting corrosion, the effect of flow velocity on corrosion is complex. When flow velocity is elevated, on one hand, corrosion might be further exacerbated on account of sufficient oxygen supply and dissolution of the underlying metal caused by accelerated mechanical damage; on the other hand, corrosion may also be retarded due to the replenishment of inhibitors (corrosion products) to some dead spaces (grooves or pores), which hinder further corrosion [25]. Thus, in the low flow velocity range, the competition between the above-mentioned two effects approaches a balance and the corrosion rate increases slowly with increasing flow velocity. However, when flow velocity exceeds a critical value, the protruding mechanical action overshadows the inhibitors replenishing, and the materials are allowed to deteriorate at a higher corrosion rate, for instance, the corrosion at 2 ms^−^^1^.

Evidently, temperature imposes a greater and nearly liner impact in promoting intergranular and pitting corrosion than velocity within 60 °C. Increase in temperature caused the acceleration of ion mobility and a corresponding rise in electrochemical reaction activity. Despite this, there is a critical corrosion temperature for materials. For instance, the corrosion rate of iron begins to decrease above 80 °C [26], while the corrosion rate of P110 steel reaches the maximum at 60 °C and then decreases [27]. For tungsten and W–Cr–C coatings in this study, the critical temperatures should be higher than 60 °C. Therefore, in the service temperature range, temperature promoted the corrosion rate of both samples.

In the test duration, W–Cr–C endures less weight loss and corrosion rate than intergranular corrosion of tungsten, showing an expected protective role. However, it is worth pointing out that pitting corrosion occurred on the W–Cr–C layer. With an increase in time, the pores will grow gradually, and when the pores stretch to the W_2_C layer, the columnar crystal structure of W_2_C will lead the corrosion mechanism to change into intergranular corrosion, which is not conducive to protecting the substrate. Furthermore, the W_2_C phase is less stable than WC in neutral solution [28]. Therefore, changing the microstructure of the W_2_C layer and increasing the proportion of the W–Cr–C layer by adjusting the carburizing parameters is the topic of ongoing work.

## 5. Conclusions

This work studied the corrosion behavior of tungsten, with and without W–Cr–C cladding prepared by the SPS technique, in a flowing water system, and the main results can be concluded as the follows:

(1) The commercial bare tungsten showed an electrochemically-driven type of intergranular corrosion, with a larger corrosion rate due to impurities segregated at the grain boundaries. After W–Cr–C cladding, the corrosion rate of tungsten dropped significantly due to the change of corrosion type from intergranular corrosion to pitting corrosion.
(2) The simultaneous increase of water temperature and flow velocity accelerated corrosion of the samples with and without the W–Cr–C cladding. The corrosion rate of the W–Cr–C coating was slower than that of tungsten under the same parameters, demonstrating its better corrosion-erosion resistance than that of pure tungsten.

## Figures and Tables

**Table 1:** The impurities with concentration over 0.001 wt.% in tungsten bulk.

Mo	Fe	Al	Si	P	C	N	O	W
0.009	0.001	0.001	0.001	0.001	0.001	0.001	0.0014	Bal

**Table 2:** The parameters set in the flowing water test.

Sample	Temperature (±3 °C)	Velocity (±0.1 ms^−1^)	Characterization
Tungsten and W–Cr–C coated tungsten	20 60	2 1, 1.5, 2	Corrosion rate and Morphology
